# Scrofula and Its Gland Diseases

**Published:** 1883-06

**Authors:** 


**Scrofula and its Gland Diseases.** An introduction to the general pathology of Scrofula, with an account of the histology, diagnosis and treatment of its glandular affections. By FREDERICK TREVES, F. R. C. S., England, Assistant Surgeon at the London Hospital. Philadelphia: H. C. Lea's Son & Co. 1883. Price 10c.

This is another of that remarkable series of medical books published by Lea and others at the ridiculously low price of ten cents. Nearly all of the volumes published, so far, have been of sterling merit. The one before us is of great value to every practitioner, as the most recent facts in connection with this widespread disease are well brought out, and a careful and wise consideration is given of the recent theories that have been expressed as to its nature and relationships.

